# Advances in Carbon-Based Microfiber Electrodes for Neural Interfacing

**DOI:** 10.3389/fnins.2021.658703

**Published:** 2021-04-12

**Authors:** Maryam Hejazi, Wei Tong, Michael R. Ibbotson, Steven Prawer, David J. Garrett

**Affiliations:** ^1^School of Physics, The University of Melbourne, Parkville, VIC, Australia; ^2^National Vision Research Institute, The Australian College of Optometry, Carlton, VIC, Australia; ^3^Department of Optometry and Vision Sciences, The University of Melbourne, Parkville, VIC, Australia; ^4^School of Engineering, RMIT University, Melbourne, VIC, Australia

**Keywords:** neural interface, carbon-based microfiber, stimulation, recording, fabrication

## Abstract

Neural interfacing devices using penetrating microelectrode arrays have emerged as an important tool in both neuroscience research and medical applications. These implantable microelectrode arrays enable communication between man-made devices and the nervous system by detecting and/or evoking neuronal activities. Recent years have seen rapid development of electrodes fabricated using flexible, ultrathin carbon-based microfibers. Compared to electrodes fabricated using rigid materials and larger cross-sections, these microfiber electrodes have been shown to reduce foreign body responses after implantation, with improved signal-to-noise ratio for neural recording and enhanced resolution for neural stimulation. Here, we review recent progress of carbon-based microfiber electrodes in terms of material composition and fabrication technology. The remaining challenges and future directions for development of these arrays will also be discussed. Overall, these microfiber electrodes are expected to improve the longevity and reliability of neural interfacing devices.

## Introduction

Neural interfaces based on microelectrode arrays (MEA) have broadened our understanding of the brain and have shown promise for treating neurological disorders due to diseases and injuries (McCarthy et al., 2011). Some examples include the cardiac pacemaker, cochlear implant, retinal prothesis, spinal cord stimulator for pain control and deep brain stimulator for epilepsy and Parkinson’s disease (Hu et al., 2016; Agarwal et al., 2017; Mulpuru et al., 2017; Naples and Ruckenstein, 2020; Tong et al., 2020). To interface with neurons, microelectrodes are implanted in the nervous system to monitor and/or modulate neural activity (Kita and Wightman, 2008; Jacobs et al., 2014; Thompson et al., 2016). Compared to non-penetrating surface electrodes, such as in electrocorticography (ECoG) and electroencephalogram (EEG), penetrating microelectrodes can communicate with neurons with higher spatial and temporal resolution due to the closer distance between the electrodes and target neural tissue (Wang et al., 2017; Konerding et al., 2018).

Over the past few decades, various types of penetrating microelectrodes have been developed. Microelectrodes fabricated using metal wires were the first used in neural recording (Hubel, 1957; Hubel and Wiesel, 1962; Wise et al., 1970; McNaughton et al., 1983; Campbell et al., 1990). These electrodes are normally based on insulated 10–200 μm diameter metal wires with an uninsulated tip used to capture the biopotential from neurons in the vicinity of the tip (Szostak et al., 2017). Different metal wires have been used, including tungsten (W) (Shuang et al., 2020), platinum (Pt) (Rose and Robblee, 1990; Wei et al., 2015), platinum/iridium (PtIr) (Zheng, 2017; Obaid et al., 2020) and titanium (Ti) (McCarthy et al., 2011). Tungsten electrodes enabled the first recording of electrical activity from individual neurons in cat brain, which later led to Nobel Prize winning work expanding our understanding of the visual cortex (Hubel and Wiesel, 1962). One limitation of metal wire electrodes is the difficulty involved in assembly into electrode arrays for simultaneous stimulation or recording from multiple regions (Pei and Chen, 2018). This fabrication challenge has been addressed by the development of silicon-based electrodes, such as the Utah (Blackrock/Cyberkinetics) (Campbell et al., 1990) and Michigan (NeuroNexus) arrays (Wise et al., 1970). A standard Utah array consists of up to 100 conical needle shaped electrodes (Choi et al., 2018), which are rigid and have diameters of 80 μm at the base tapering to a tip. Utah arrays are primarily used in large animals, especially non-human primates (Choi et al., 2018), and remain a common choice for obtaining high dimensionality recording of spiking neural activity in clinical and basic neuroscience research (Cody et al., 2018). Compared to Utah arrays, NeuroNexus probes are thinner and have more flexible silicon shanks. Each shank has multiple iridium electrode sites positioned along it (Wise et al., 1970; Takmakov et al., 2015; Ferguson et al., 2019). NeuroNexus probes are more often used in small animals (e.g., rodents and cats). NeuroNexus probes are longer than those in the Utah array (2–15 mm vs. 0.5–1.5 mm) (Choi et al., 2018), therefore they are more suitable for capturing recordings from deeper regions (Choi et al., 2018; Almasi et al., 2020).

Although all the electrodes mentioned above are suitable for acute studies, they exhibit limited lifetimes after implantation, which restrict their chronic and clinical applications (Leber et al., 2017). There are at least two major reasons that may account for device failure. First, the implantation of the electrodes has been found to evoke inflammatory tissue response in the host body. The inflammatory tissue response is initiated by insertion damage and persists due to the mismatch of chemical and physical properties between the electrodes and the surrounding tissues. An inflammatory tissue response leads to neuronal death and glial scar formation, which reduces the strength of neural signals and also leads to changes in electrode properties (Gulino et al., 2019). Second, the lifetime of the implanted devices is limited by material instability. For example, cracking and delamination has previously been observed near the electrode sites in NeuroNexus probes after long-term implantation (Kozai et al., 2015). The electrochemical properties of electrode materials have also been found to change following repetitive stimulation (Kozai et al., 2015). Other factors that contribute to the longer term instability of electrode performance include electrode site corrosion, as in tungsten electrodes (Sankar et al., 2014), and electrode material degradation, as in poly(3,4-ethylenedioxythiophene) polystyrene sulfonate (PEDOT:PSS) (Cameron and Skabara, 2020). One strategy to overcome the issue of the inflammatory tissue responses is to use thin polymeric materials, such as polyimide (Castagnola et al., 2013, 2014), Parylene-C (Agorelius et al., 2015) and SU-8 (Xie et al., 2015; Luan et al., 2017; Liu, 2018; Zhao et al., 2019) as the substrate for flexible MEAs to match the soft nature of the brain and minimize perpetual machinal trauma and inflammation. These flexible probes have been demonstrated to better integrate with the neural tissue, with potential to record single unit neural signals for months.

In addition to improved longevity, the next generation of neural interfaces require the use of electrodes with enhanced functionality. In these devices, the electrodes should support closed-loop operation by providing reliable and comprehensive information via recording, and also use the recorded information as the feedback to inform and precisely modulate neural stimulation. Real-time feedback signals from such a bi-directional system can improve the performance of neural interfaces in two ways: by allowing real-time correction of errors and by activating a learning process in the areas involved in the loop (Angotzi et al., 2014). Therefore, these closed-loop interfaces will enable a higher-level understanding of neural functions and advance the development of novel therapies (Zhou et al., 2019). The trend of device miniaturization makes it highly desirable that the same electrodes are capable of both neural stimulation and recording. This is a significant material sciences challenge as the electrode materials need to possess a wide range of electrochemical properties if they are to be used for both recording and stimulation. Examples of clinical applications that may benefit from the use of closed-loop system include epilepsy prediction and treatment via electrical stimulation of spinal cord (Berenyi et al., 2012; Pais-Vieira et al., 2016), deep brain stimulation for Parkinson’s diseases treatment (Fleming et al., 2020) and tremor suppression (Opri et al., 2020). In these applications, the use of closed-loop system has been shown with similar or even better clinical efficacy compared to the use of open-loop system, while having a consistent reduction in energy requirement (Fleming et al., 2020; Opri et al., 2020).

An alternative to existing metal or silicon-based microelectrodes is microfiber electrodes, which are mostly fabricated from carbon-based materials such as carbon fibers (CFs), carbon nanotubes (CNTs) and graphene. These carbon-based microfibers, with low micron dimensions, softer surfaces, improved flexibility, and adjustable electrochemical properties, can solve a lot of challenges associated with other electrode designs. Overall, these microfiber electrodes have been shown to remain viable for longer periods in chronic applications due to minimal tissue responses as a result of reduced electrode sizes and better compliance with the surrounding tissue (Stice et al., 2007; Guitchounts et al., 2013; Karumbaiah et al., 2013). With proper surface modifications, many of these microfiber electrodes can enable closed-loop function as they exhibit appropriate properties for high precision neural recording and stimulation. They have also been demonstrated with better stability when used for neural interfacing (Vomero et al., 2017; Nimbalkar et al., 2018; Vahidi et al., 2020). Among them, CF microelectrodes with small cross sections have been used for detection of neurotransmitters such as dopamine and serotine for over three decades in the brain using fast-scan cyclic voltammetry (Robinson et al., 2003; Dankoski and Wightman, 2013; Taylor et al., 2015; Castagnola et al., 2020). However, only recently they have been used for neural recording (Kozai et al., 2012, 2016; Patel et al., 2015, 2016). Most of these research uses CF microelectrodes in the form of single fibers (Kozai et al., 2012; Apollo et al., 2015; Vitale et al., 2015), but the techniques for assembling these fibers into high-density microelectrode arrays are also under development (Guitchounts et al., 2013; Patel et al., 2015, 2016, 2020; Gillis et al., 2018; Massey et al., 2019; Guitchounts and Cox, 2020).

This review focuses on recent progress in developing carbon-based microfiber electrodes for neural stimulation and recording. We will first discuss in detail the advantages of using carbon-based microfibers for neural interfacing. We will then summarize the electrode materials that have been used so far and review current technologies for integrating individual microfibers into high density arrays. Finally, remaining challenges and future trends will be discussed.

## Advantages of Carbon-Based Microfiber Electrodes

**FIGURE 1:**
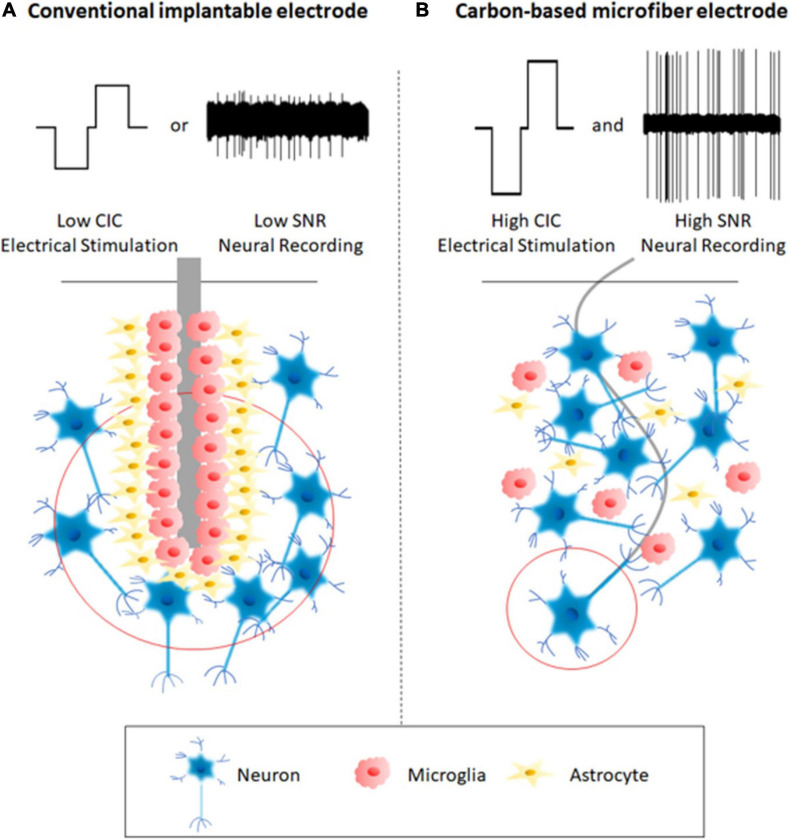
A comparison between a conventional implantable electrode **(A)** and a carbon-based microfiber electrode **(B)**. Schematic A shows that the implantation of a conventional implantable electrode can lead to severe tissue responses and glial scar formation around the electrodes, which account for the device’s instability and even failure. The conventional electrodes communicate with neurons with low spatial accuracy because of the large electric field, which encompasses a large number of neurons. These conventional electrodes mostly function in one way, either neural stimulation or recording, and therefore are not suitable for closed-loop operation. In contrast, Schematic B shows that the use of carbon-based microfiber electrodes reduces adverse tissue responses by eliminating glial scar formation, targets single neurons due to the localized electric field (red circle), provides higher charge injection capacity (CIC) for electrical stimulation, and leads to a higher signal-to-noise ratio (SNR) during neural recording.

Compared to conventional microelectrodes fabricated using metal wires and silicon technologies, carbon-based microfiber electrodes share some common characteristics. They have smaller cross-sections, exhibit better mechanical properties, and enhanced electrochemical characteristics. Overall, these features contribute to the improved longevity and functionality of the electrodes, as a result of their reduced adverse tissue response and better performance in neural stimulation and recording. Figure 1 illustrates the advantages of carbon-based microfiber electrodes over conventional implantable microelectrodes.

### Minimal Tissue Response

The first and foremost challenge associated with micro MEA development is their inconsistent performance during long-term applications, mostly due to the adverse tissue response after electrode implantation (Ghane-Motlagh and Sawan, 2013). The adverse tissue response begins at electrode insertion, which causes physical trauma as the electrode displaces and damages the blood–brain barrier (BBB), neural cells and the extracellular matrix (ECM) on its path to the intended target (Sommakia et al., 2014). In weeks following implantation, a fibrous envelope gradually forms around the electrodes, mostly composed of activated microglia and astrocytes (Figure 1A). This is commonly referred to as the glial scar (Harris and Tyler, 2013; Sridharan et al., 2013). The mismatch of the chemical and physical properties between the electrode and brain tissue results in sustained inflammation and neural degeneration (Kotzar et al., 2002; Polikov et al., 2005; Spearman et al., 2017). The inflammatory process hinders stimulation and recording of neuronal cells and contributes to device failure as a result of electrode degradation (Geddes and Roeder, 2003). In this section, we will focus our discussion on the advantages of using carbon-based microfibers as the electrode materials. However, it is often required to assemble these microfibers into high-density MEAs for many applications. The design of MEA, such as the choice of substrate materials, can also have an impact on the tissue response, which will be discussed in see section “Electrode Alignment and Assembling.”

Carbon-based microfibers have several merits that help to avoid device failure by minimizing tissue response. First, their small cross-sections enable a significant reduction in footprint that minimizes insertion damage. As shown in Table 1, most carbon-based microfibers have cylindrical shapes with a diameter below 30 μm. In comparison, although metal wire electrodes and Utah arrays have small tips, they have a conical shape with the diameters of the base much larger, up to 200 μm (Szostak et al., 2017) and 80 μm (Choi et al., 2018), respectively. A standard NeuroNexus probe has a cross-section as large as 1,845 μm^2^ with a thickness of 15 μm and a width at the base of 123 μm (Deku et al., 2018a). The rupture of the BBB is known as the first and one of the most critical events occurring during electrode insertion (Bennett et al., 2018). Previous research has shown that BBB rupture is heavily involved in the triggering of biochemical pathways responsible for neuronal degeneration and glial activation (Bennett et al., 2018). Plasma proteins released from the BBB can also accumulate at the injury site and be adsorbed at the electrode surface which alter electrode properties and impair performance. The smaller feature size of carbon-based microfiber electrodes has therefore been suggested as a key factor to minimize tissue response by reducing damage to the BBB (Kozai et al., 2012).

Many carbon-based microfibers have been confirmed as biocompatible and biostable, both *in vitro* and *in vivo* (Smart et al., 2006; Chang et al., 2011; Kim et al., 2013; Guo et al., 2015; Wang et al., 2019; Hejazi et al., 2020b). Biocompatibility refers to biological “harmlessness,” or, alternatively, how well a living organism tolerates and survives the implant without triggering unacceptable reactions or changes (Gunter et al., 2019). Biostability means that the implant is not susceptible to degradation due to the action of biological fluids, proteases, macrophages or any substances of metabolism (Marin and Fernandez, 2010). The use of biocompatible and biostable materials is expected to improve neuronal and device survival and reduce glial activation around the electrodes (Wang et al., 2018; Park et al., 2019). To assess the biocompatibility and biostability of electrode materials, *in vitro* tests use cultured cells to study their impact on cell survival, reproduction and morphologies. Commonly used models for assessing neural electrodes include primary cultures using cortical (Fan et al., 2020) and hippocampal neurons (Beach et al., 2020), and neuroblastoma cell lines such as N2a (Kim et al., 2012), PC12 (Carnicer-Lombarte et al., 2017), and SH-SY5Y (Yoon et al., 2020). Studies have focused on their impact on neuronal survival, neurite outgrowth and neurite network function (Gulino et al., 2019). As glial cells play important roles in adverse tissue response, many recent studies also investigate the effect of electrode materials on glial cells using cultures containing microglia and astrocytes (Park et al., 2001; Watson et al., 2017; Goshi et al., 2020). *In vivo* studies are normally performed by chronically implanting the electrodes in the brain. These studies can be divided into passive (no stimulation) and active (stimulation) studies. Passive studies evaluate electrode insertion trauma, electrode biocompatibility and the micromotion effects on both implant and tissue, which is relevant to the mechanical properties discussed in the next paragraph. Chronic active studies are designed to evaluate the safety and function of the electrodes which reflect both material biocompatibility and biostability (Shepherd et al., 2018; Gunter et al., 2019).

**TABLE 1:** Commonly used materials and carbon-based microfibers for neural stimulation and recording.

	Name	Dimension		Mechanical properties		Electrochemical properties				Biological performances		References
					
		Electrode shank size (μm)	Electrode site geometric surface area (μm^2^)	Young’s moduls (GPa)	Flexural rigidity (N.m^2^) or other	CIC (mC/cm^2^)	CSC (mC/cm^2^)	Water Window (V)	Impedance at 1 kHz (kΩ)	Stimulation	Recording	
**Silicon- based MEA**	Utah array (Electrode Materials: Pt or iridium oxide)	tip diameter: 25.4; base diameter: 80	2,000	165	—	—	∼36 (Iro)	−0.6–0.8 (IrO)	50–60	—	*In vivo* acute and chronic (5.75 years) recording in motor or premotor cortex in Rhesus macaques of monkeys	Barrese et al., 2013; Black et al., 2018; Choi et al., 2018
	NeuroNexus probe (Electrode Material: SIROF)	thickness: 15; width: 123	1,845	165	5.7 × 10^–10^	5.2	19.4 ± 2.4	−0.6–0.8	88.5	—	*In vivo* acute recording in rat motor cortex from SU	Deku et al., 2018a
**Metal wires**	tungsten	Cylinder diameter: 50	∼2,300	390	1.2 × 10^–7^	—	—	—	40–150	—	*In vivo* chronic recording in rat cortex and hippocampus for 4 months	Ward et al., 2009; Sankar et al., 2014; Shuang et al., 2020
	platinum	Cylinder diameter: 15	7,850	47	—	0.2	1.2	−0.6–0.8	18	—	*In vivo* acute recording of LFP in rat striatum	Apollo et al., 2015; Wei et al., 2015; Wang et al., 2019
	platinum/iridium	Cylinder diameter: 15	78.5	233	(1.23 ± 0.64) × 10^–10^	0.13	8	−0.6–0.8	200 ± 27	—	*Ex vivo* recording in rat retina; *in vivo* acute recording in deep cortical and subcortical regions in mice; *in vivo* chronic recording in motor cortex and striatum of awake moving mice for 14 days.	Venkatraman et al., 2011; Zheng, 2017; Obaid et al., 2020
**Carbon-based fibers**	bare CF	Cylinder diameter: 5 or 7	38 or 58.1	234	2.7 × 10^–11^	0.105 ± 0.067	—	−0.6–0.4	hundreds of KΩ	—	*In vivo* acute recording in rat motor cortex	Kozai et al., 2012; Massey et al., 2019; Hejazi et al., 2020a
	bare CF	Cylinder diameter: 4.5	—	380	—	—	—	—	median = 1,000	—	*In vivo* chronic recording in zebra finches for up to 107 days	Guitchounts et al., 2013
	PEDOT:PSS-co-MA coated CF	—	1076.47 or 5472.47	—	—	96–192	—	−0.9–0.4	5	*Ex vivo* stimulation in rat at the cervical spinal cord	—	Vara and Collazos-Castro, 2019
	PEDOT:PSS coated CF	Cylinder diameter:7	58.1	—	—	—	—	−0.6–0.8	up to about 100	—	*In vivo* chronic recording both SU action potentials and LFPs chronically in the mice visual cortex for 5 weeks	Kozai et al., 2012
	PEDOT: pTS coated CF	Cylinder diameter: 8.4	36.3	—	—	18.5 ± 2.6	—	—	118 ± 28 or 117.9 ± 28.4	—	*In vivo* chronic recording in rat cortex for 31 days; *In vivo* chronic recording in rat motor cortex up to 154 days	Patel et al., 2015, 2016
	PEDOT:TFP coated CF	Cylinder diameter: 4.5	—	—	—	—	—	—	170 ± 860	—	*In vivo* chronic recording of both spontaneous and visual stimuli evoked activities from rat visual cortex for 55 days	Guitchounts and Cox, 2020
	Iridium oxide film (EIROF) coated CF	Cylinder diameter: 8.5–12.5	∼385 or 600	—	—	17	∼25	−0.6–0.6	57 or ∼100	*Ex vivo* stimulation of in zebra finche tracheo syringeal nerve	*In vivo* acute recording from the tracheo syringeal nerve in zebra finches	Deku et al., 2018b; Gillis et al., 2018
	N-UNCD coated CF	Cylinder diameter: 10	3,218	—	—	7.09 ± 3.65	—	−1.8–1.2	25	*Ex vivo* stimulation of rat retina	*Ex vivo* recording from rat retina; *in vivo* acute recording in wallaby visual cortex	Hejazi et al., 2020a
	B-CNW coated CF	Cylinder diameter: 10	3,218	—	—	7.82 ± 0.35	—	−1.8–1.2	29.95 ± 13.53	*Ex vivo* stimulation of rat retina	*In vivo* acute recording in wallaby visual cortex	Hejazi et al., 2020b
	CNT fiber produced by wet spinning	Cylinder diameter: ∼50	1,450	—	—	6.52	—	−1.5–1.5	11.2 ± 7.6	*In vivo* chronic stimulation of parkinsonian rat motor cortex	*In vivo* chronic recording from rat cortex for 3 weeks	Vitale et al., 2015
	CNT fiber produced by dry spinning	Cylinder diameter: 5–20 or 20–100	—	9.7 ± 0.5	bending stiffness = 8.16 × 10 ^3^ or 1.58 × 10^2^ nN.m	3.52 ± 0.15 or 5.04 ± 0.22 (after acid nitric treatment)	278.21 ± 5.42	-0.6- 0.8	279.96 ± 32.08 or 41.95 ± 3.62 (after acid nitric treatment)	—	*In vivo* acute and chronic recording in rat ventral posteromedial nucleus of the thalamus and primary somatosensory cortex up to 5 months; *In vivo* chronic recording in mice cortex for 4 weeks	Xu and Gao, 2015; Lu et al., 2019; Tang et al., 2020
	graphene encapsulated copper microwires	Cylinder diameter: 100	—	—	—	—	—	—	∼100	—	*In vivo* acute and chronic recording of LFPs and SU action potentials in rat hippocampus for at least 4 weeks	Zhao et al., 2016
	liquid crystal graphene oxide (LCGO) fiber	Cylinder diameter: 40–50	—	11.2	—	14 ± 0.9	—	−1–0.9	∼5	*Ex vivo* stimulation of rat retina	*In vivo* acute recording in feline visual cortex	Apollo et al., 2015; Xu and Gao, 2015.
	platinum coated LCGO fiber	Cylinder diameter: ∼20 or ∼40	169 ± 25 or 749 ± 93	—	—	10.34	—	−1–0.9	—	—	*In vivo* acute SU recording in rat cerebral cortex	Wang et al., 2019
	graphene fiber	Cylinder diameter: 75	—	2–3	—	10.1 ± 2.25	889.8 ± 158.0	−1.5–1.3	15.1 ± 3.67	*In vivo* deep brain stimulation in a behaving Parkinson rat model	—	Zhao et al., 2020
	polyethylene (CPE) and 5 wt% graphite fiber composites	Cylinder diameter: 200	—	—	stiffness = 76.1–83.5 N/m	—	—	—	1,310 ± 270	*In vivo* optical stimulation of mouse cortex and hippocampus	*In vivo* chronic recording in mouse cortex and hippocampus for 3 months	Park et al., 2017

Carbon-based microfibers possess favorable mechanical properties over many conventional electrode materials, contributing to minimal tissue response after implantation. The mechanical properties of carbon-based microfibers and other electrode materials are summarized in Table 1. While the Young’s modulus of brain tissue is below 15 kPa, silicon-based arrays and metal electrodes show much larger Young’s modulus which are in the range of 107–390 GPa (Woeppel et al., 2017). The mechanical mismatch between the electrodes and brain tissue leads to stress at the electrode/tissue interface and induces the chronic inflammatory response (Shuang et al., 2020). In contrast, many carbon-based materials are softer, with smaller Young’s modulus, such as 11.2 GPa in liquid crystal graphene oxide (LCGO) fibers (Xu and Gao, 2015). Another characteristic of carbon-based microfibers is that they normally exhibit reduced bending stiffness and better flexibility. Bending stiffness, also known as flexural rigidity, depends upon both the geometry and material composition (Deku et al., 2018a). The bending stiffness *D* is calculated by multiplying Young’s modulus *E* and moment of inertia *I* (Deku et al., 2018a) as shown in Eq. 1:

(1) D=E⁢I

A fiber structured electrode can be modeled as a core-shell cylindrical probe, and therefore its bending stiffness *D* can be calculated according to the Eq. 2 (Lu et al., 2019):

(2) $$D=E_{c\,o\,r\,e}\,\frac{\pi \,d_{i}^{4}}{64}+E_{s\,h\,e\,l\,l}\,\frac{\pi \,d_{0}^{4}}{64}\,\left[1-{\left(\frac{d_{i}}{d_{0}}\right)}^{4}\right]$$

where *E*_*core*_ is the Young’s modulus of the conductive fiber, *E*_*shell*_ is the Young’s modulus of the insulated coating layer, *d*_*i*_ represents the diameter of the conductive fiber, *d*_*o*_ represents the total diameter (including the insulation layer) of the core-shell cylindrical microelectrodes. According to Eq. 2, fibers with smaller cross section tend to have a smaller bending stiffness. Therefore, it is possible to create flexible neural implants using materials with high Young’s modulus if the geometric cross-section is greatly reduced. Flexible implants cause less micromotion-induced damage because of their shock absorption and vibration dampening properties (Gillis et al., 2018). A drawback of flexible electrodes is that the electrode insertion may be difficult. To address this challenge, different strategies have been developed to facilitate the insertion of flexible electrodes. One example is to temporarily improve the electrode stiffness by coating the flexible fibers with materials such as silk and sucrose, which dissolve into the surrounding tissue after electrode insertion (Tien et al., 2013; Apollo et al., 2015). The insertion techniques will be discussed further in Section “Insertion Techniques”.

### High Precision Neural Stimulation and Recording

The next generation of neural interfaces requires closed-loop operation, in which the same electrodes are expected to talk to the nervous system in both directions by performing electrical stimulation and recording. During neural stimulation and recording, it is also desired that the electrodes can communicate with single or a small group of neurons with high spatiotemporal resolution. Small electrodes with cellular dimensions, such as carbon-based microfibers, have advantages for high precision neural stimulation and recording as the electric fields become more localized when electrode sizes are reduced (Figure 1). Electrochemical properties are important parameters to consider when designing microelectrodes. The great potential for surface modification of carbon materials makes it possible to fabricate electrodes with different electrochemical properties capable of fulfilling the design of bi-directional neural interfacing.

#### Neural Stimulation

During neural stimulation, electrode materials are required to inject sufficient charge into the neural tissue to evoke neural activities without damaging the electrodes or the surrounding tissue. Charge injection capacity (CIC) is a figure of merit used in neural stimulation research to describe the maximum amount of charge that can be safely injected during a single stimulation pulse before the water-window is exceeded (Cogan, 2008). Water window refers to the safe potential range in which the electrode is stable (Cogan, 2008). Water windows can be measured using cyclic voltammetry with a three-electrode setup in saline solution and they differ between different materials (Table 1).

There are several methods of measuring CIC. The most commonly used technique is voltage transient measurement, in which the voltage transients are measured while constant current stimulation pulses are applied on the electrodes (Cogan, 2008). The voltage transients are analyzed to determine the maximum charge that can be injected when both the most negative (E_*mc*_) and most positive (E_*ma*_) potentials across the electrode-electrolyte interface are within the water window (Cogan, 2008). Normally, CIC is dependent on the pulse duration and it increases when longer pulses are used. Recent research also shows the relationship between CIC and the geometric surface area (GSA) of the electrode that CIC increases with GSA size (Ganji et al., 2017). Som publications also report CIC estimated by measuring the double layer capacitance at the electrode/solution interface (Garrett et al., 2012; Hejazi et al., 2020a, b). Here, CIC is calculated according to Eq. 3,

(3) $$C\,I\,C=\left(C_{d\,l}\times V_{m}\right)/G\,S\,A$$

where C_*dl*_ is the specific electrochemical capacitance, V_*m*_ is the voltage threshold for electrolysis of water and GSA is the geometric surface area of the electrode exposed to the solution (Garrett et al., 2012). C_*dl*_ can be estimated either from cyclic voltammetry or by fitting an equivalent electrical circuit model to electrochemical impedance spectroscopy (EIS) data (Apollo et al., 2015; Hejazi et al., 2020a).

In addition to CIC, many publications also use charge storage capacity (CSC) for comparing stimulation electrode performance (Ganji et al., 2017). CSC is calculated according to Eq. 4:

(4) $$C\,S\,C=Q_{s\,t\,o\,r\,a\,g\,e}/G\,S\,A$$

where Q _*storage*_ is cathodic or anodic charge storage calculated from the time integral of the cathodic (negative) or anodic (positive) current in cyclic voltammetry at a specific sweep rate over a potential range within the water window (Ganji et al., 2017). Compared to CIC, CSC is measured using lower voltage scanning rates, and the value of CSC from one material is normally larger than that of CIC. Carbon-based microfibers typically show higher CIC and CSC values due to their higher conductivity and larger effective surface area than many other electrode materials (Table 1).

Long-term neural stimulation requires the electrode to exhibit stable properties during repetitive stimulation. There are several methods for evaluating the stability of the stimulation electrodes. The first method is to monitor the electrode properties during and after continuous stimulation with biphasic pulses (Hejazi et al., 2020b). The properties that are compared before and after several million pulses include CIC values, electrode impedances and the electrode surface morphology. Voltage cycling tests provide another method for studying the electrode stability, in which both CSC values and surface morphologies are compared after thousands (1,000–17,000) of repetitive CV cycles (Peixoto et al., 2009; Venkatraman et al., 2011; Hejazi et al., 2020b). Many carbon-based microfibers have been shown to exhibit good stability after repetitive stimulation (Bennet et al., 2016; Hejazi et al., 2020b).

#### Neural Recording

Neural signals recorded extracellularly using implanted electrodes can be analyzed to extract at least two different types of voltage signals: local field potentials (LFPs) and single-unit (SU) action potentials. LFPs reflect collective transmembrane currents from multiple neurons and therefore the activity of a local neural network (Burns et al., 2010; Herreras, 2016). LFP signals are normally stable over time, but at the expense of decreased spatiotemporal resolution. SU action potentials represent the activity from individual neurons adjacent to the recording electrode tips. They provide better spatiotemporal resolution than LFPs and are important for understanding the inner working of the brain (Sharma et al., 2015).

To obtain high-quality SU action potentials, the electrodes are required to record with high signal-to-noise ratio (SNR) and detect very small amplitudes of action potentials against a noisy background. The most commonly used parameter for comparing different recording electrodes is their electrochemical impedance at 1 kHz, which can be measured using EIS. Low impedance allows for low noise, implicating an improved SNR (Nick et al., 2012; Kim et al., 2017). Ideally the impedance for a recording electrode should be less than hundreds of kΩ for low thermal noise and a high SNR of neural signals (Kim et al., 2017). The SNR can also be influenced by the GSA of the electrodes and smaller recording sites have been shown to enhance the sensitivity and spatial selectivity of recording (Castagnola et al., 2014). It was found that recording amplitude of SU decreases rapidly for electrode surface areas larger than 100 μm^2^, therefore electrodes smaller than 100 μm^2^ are ideal for detecting SU activities (Hill et al., 2018). However, electrode impedance increases when the electrode size decreases. Furthermore, for electrodes smaller than 10 μm, the noise and signal attenuation depend more on the electrode impedance than on electrode size (Viswam et al., 2019). One commonly used strategy to lower the impedance of electrodes is to use materials with large effective surface areas. Carbon-based microfibers, with cross-sectional diameters smaller than 30 μm and large effective surface areas, have lower electrochemical impedance than many other materials, and therefore have been found to record with higher SNRs (Table 1).

The quality of electrode recording can be assessed using both *ex vivo* and *in vivo* biological models. *Ex vivo* models include explanted rat retina and brain slices. The retina, which processes visual information and sends it to the brain, is an excellent model for studying neural circuitry (Hong et al., 2018). In this model, the electrodes are normally placed in direct contact with the retinal ganglion cells (RGCs) in the retina. Since the retina is light sensitive, a light source is switched on and off to elicit neural activity for recording (Sim et al., 2014; Hejazi et al., 2020a). Brain slice preparations have been used to study the electrical behavior of individual neurons and the function of neural systems (Suter et al., 1999), as the neurons in this model can reflect both electrophysiological and pharmacological responses similar to those in the intact nervous system (Suter et al., 1999). Both thick (∼500 μm) and thin (∼150–350 μm) slices have been developed for *ex vivo* recording. In the thick slice preparation, many local connections between neurons are maintained, making it useful for examining intrinsic membrane properties and drug effects in relatively intact cells and for studying local synaptic circuits. Thin slice preparations allow neurons to be visualized at high magnification under a compound microscope for patch-clamp recordings (Suter et al., 1999). *In vivo* models include either acute or chronic recording from cortex and hippocampus, whose neurons are involved in physiological central nervous system (CNS) processes such as learning and memory (Yin et al., 2016). In those models, the electrodes are implanted in the cortex or hippocampus regions of the brain of either anesthetized or awake animals, and spiking activities are collected during a short term of several minutes or hours, or for a longer period from several weeks up to months or years.

## Carbon-Based Microfibers for Neural Interfacing

**FIGURE 2:**
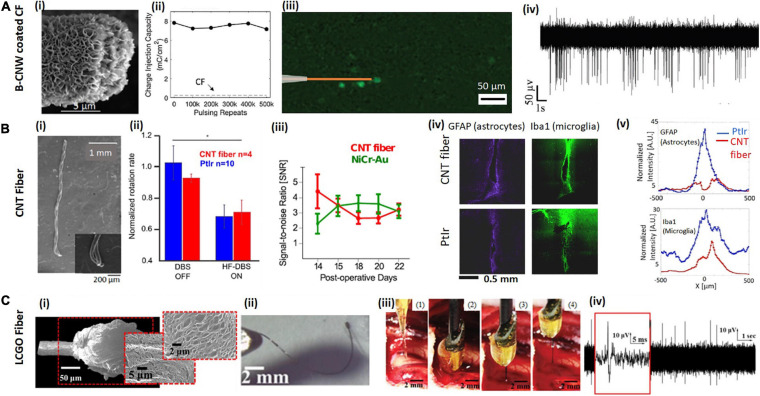
Examples of carbon-based microfibers for neural interfacing. **(A)** B-CNW coated CF. **(Ai)** SEM image of a B-CNW coated CF single-fiber electrode. **(Aii)** B-CNW coated CFs show good stability after repeated biphasic stimulation and the CIC of a B-CNW coated electrode (solid line) remains significantly higher than that of a bare CF electrode (dash line). **(Aiii)** B-CNW coated electrodes elicit localized response from retinal ganglion cells (RGCs) in the explanted rat retina. **(Aiv)** *In vivo* acute recording from wallaby visual cortex shows a high SNR Reproduced from Hejazi et al. (2020b) with permission from the copyright holder. **(B)** CNT fiber. **(Bi)** SEM images of two-channel CNT fiber microelectrodes show the fibers with good flexibility. **(Bii)** CNT fiber electrodes show comparable efficacy with PtIr electrodes in deep brain stimulation of Parkinsonian rats. The average normalized rotation rates of rats implanted with CNT fiber microelectrodes are similar with that implanted with PtIr electrodes. **(Biii)** Time evolution of the SNR over the 2 weeks of recording sessions using CNT fibers and NiCr-Au control electrodes. After initial fluctuations caused by inflammatory response to the electrode implant, SNR reaches stable values of ∼6 SD, which confirms that CNT fibers are suitable for chronic recordings. **(Biv,v)** Fluorescence images of tissue response after 6 weeks of implant with a CNT fiber, compared to a PtIr electrode implanted contralaterally. Panels show tissue labeled for astrocytes and microglia and Fluorescence intensity profiles at increasing lateral distance x from electrode midline: astrocytes and microglia. GFAP is abbreviation of rabbit antiglial fibrillary acidic protein and IBa1 stands for mouse anti-ionized calcium binding adaptor molecule 1. Reproducedfrom Vitale et al. (2015) with permission from the copyright holder. **(C)** Liquid crystal graphene oxide (LCGO) fiber. **(Ci)** Low and high magnification SEM images of a LCGO brush electrode after laser treatment. **(Cii)** LCGO fibers demonstrate flexibility and elastic deformation. **(Ciii)** To facilitate electrode insertion, a LCGO fiber electrode is coated in a rigid sucrose carrier needle and implanted into the feline brain, then removed from brain after 15 min of recording; sugar needle is completely dissolved. **(Civ)** LCGO electrodes can record neural activity with a high SNR. Reproduced from Apollo et al. (2015) with permission from the copyright holder.

Carbon-based microfibers fabricated into electrodes for neural stimulation and recording come mainly in three forms: carbon fibers, CNT-based fibers and graphene-based fibers. The materials and their properties are summarized in Table 1, with some examples shown in Figure 2.

### Carbon Fibers (CF)

Carbon fiber is one of the most commonly used carbon-based microfiber electrodes (Chen et al., 2020), which were first developed in the late 1970’s (Santos et al., 2008). They have low cost, and can be prepared with porous structures, therefore with large surface areas (Roeser et al., 2013). The majority of CFs are produced by heat treatment of polyacrylonitrile (PAN)-based precursors (Hung et al., 2017), which can result in fibers with moduli stiffer and stronger than steel, whilst still retaining good flexibility (Petersen, 2016; Cetinkaya et al., 2018). Microelectrodes fabricated using CFs normally have diameters between 4 and 10 μm, available with different stiffness and surface smoothness. Those CFs with extremely small size (cross-section of 60 μm^2^) need to be temporarily stiffened to assist with brain insertion. For instance, Schwerdt et al. (2018) stiffened CFs using polyethylene glycol (PEG) shuttle (0.5–1 mm thick). The PEG shuttle was incrementally dissolved just above the brain surface, so as to suspend small lengths of the probes as they were progressively lowered without deflection into the tissue. CFs have been used for neural recording both *in vitro* and *in vivo* (Kozai et al., 2012; Gillis et al., 2018; Massey et al., 2019; Guitchounts and Cox, 2020; Hejazi et al., 2020a). Due to their small dimension, CF electrodes have been found to trigger negligible immune response upon implantation, therefore they are suitable for chronic *in vivo* applications. For example (Guitchounts et al., 2013) reported the use of a CF electrode array with 16 channels for *in vivo* recording in HVC, a song motor nucleus, in singing zebra finches for up to 107 days after implantation.

Although CFs are suitable for neural recording, additional coatings are normally required to improve their CICs for neural stimulation (Deku et al., 2018b; Gillis et al., 2018; Vara and Collazos-Castro, 2019; Hejazi et al., 2020a, b). Such coatings also often lead to smaller electrochemical impedances, which therefore improve the quality of recording from the electrodes. The coating materials that have been developed include conductive polymers, iridium oxide and other carbon-based materials such as carbon nanowalls and conductive diamond.

Conductive polymers are the most widely used coating materials for CF microelectrodes. They have been developed as neural interfacing materials due to several advantages such as small Young’s modulus, high conductivity, large CIC and low electrochemical impedance (Yang et al., 2005; Pranti et al., 2017; Watanabe et al., 2017; Kim et al., 2019; Seo et al., 2019). To coat carbon fibers, PEDOT and their blends can be deposited onto the carbon fiber electrodes using electroplating. Different doped PEDOT coatings have been reported including PEDOT:PSS, PEDOT:pTS, and PEDOT:TFB (Kozai et al., 2012; Patel et al., 2015; Vara and Collazos-Castro, 2019). Kozai et al. (2012) reported that CF with a coating of PEDOT:PSS can largely decrease the impedance of the electrodes while increasing their CSC. The implanted electrodes were able to record both SU action potentials and LFPs chronically in mouse visual cortex for 5 weeks. Compared to the NeuroNexus probes, the recordings using PEDOT:PSS coated CFs showed higher SNRs and signal amplitudes, while lower levels of glial scarring were detected as indicated from histology. It was suggested that the reduced tissue response might be attributed to the smaller insertion footprint of the coated CF electrodes. In this study, microelectrodes were also coated with anti-biofouling materials, improving chronic electrode performance. The instability of conductive polymers has been suggested as one limitation for conducting polymer modified CF electrodes in chronic applications (Mandal et al., 2015; Cameron and Skabara, 2020). Patel et al. (2016) compared the stability of PEDOT:pTS and PEDOT:PSS coatings using accelerated soaking tests and measured the change of impedance over time. From their results, PEDOT:pTS showed better stability than PEDOT:PSS coating, and they subsequently used PEDOT:pTS coated CFs for chronic recording in rat motor cortex up to 154 days (Patel et al., 2016). It has also been reported that PEDOT:TFB functionalized CFs are capable of recording both spontaneous and visual stimulus evoked activities from the visual cortex of freely moving rats for 55 days (Guitchounts and Cox, 2020).

The electrodeposited iridium oxide film (EIROF) is another coating that has been used on CFs to boost their electrochemical properties (Deku et al., 2018b; Gillis et al., 2018). Electrode coatings with EIROF or sputtered iridium oxide films (SIROF) have been proposed for stable chronic neural interfaces for neural stimulation due to their large injection capacity and relatively high stability following repetitive stimulation (Cogan et al., 2009; Deku et al., 2018b; Gillis et al., 2018). Such coatings have been also previously proved biocompatible *in vitro* as they could support neural cell attachment and neurite outgrowth (Chen et al., 2019). By electrodepositing a thin layer of EIROFs, the electrode impedance of CFs was reduced by a factor of 10 and the CIC increased to more than 17 mC/cm^2^ with appropriate biasing. The coated electrodes were able to record acute SU spontaneous activities from the tracheosyringeal nerve of zebra finches and evoke responses via electrical stimulation (Gillis et al., 2018). However, iridium oxide coatings have been previously reported with poor adhesion to underlying substrates, and they may degrade under chronic aggressive stimulations due to its low structural and chemical stability (Mailley et al., 2002; Cogan et al., 2004).

The Melbourne group have demonstrated two types of carbon-based coatings to improve the performance of CF electrodes for neural interfacing, viz., nitrogen included ultrananocyrslline diamond (N-UNCD) (Hejazi et al., 2020a) and boron-doped carbon nanowalls (B-CNW) (Hejazi et al., 2020b). N-UNCD is biocompatible due to its chemical and biochemical inertness, and has previously been used as the electrode material for neural stimulation in a retinal prosthetic device for restoring vision (Garrett et al., 2012; Hadjinicolaou et al., 2012; Ganesan et al., 2014; Tong et al., 2016; Ahnood et al., 2017). It is chemically non-cytotoxic (inert) when in contact with neural tissue (Garrett et al., 2016a, b; Tong et al., 2016), and it is highly resistant to surface biofouling and chemical degradation (Bennet et al., 2013; Yang et al., 2019). We showed that after coating the CFs with N-UNCD, CIC increased 238-fold and impedance decreased by 25%. The coated electrodes were also shown to successfully evoke neural activity in explanted retina and record SU activities from visual cortex (Hejazi et al., 2020a). B-CNW coatings were also developed (Figure 2Ai), which showed similar CIC and impedance to the N-UNCD coated materials due to its large effective surface area. The B-CNW coating was demonstrated to be biocompatible, supporting the growth of cortical neurons *in vitro*. When used for neural stimulation, the B-CNW coated electrodes showed excellent stability after a repetitive pulsing test (Figure 2Aii). They were also found to result in high resolution RGC stimulation (Figure 2Aiii), and a higher SNR from *in vivo* recording (Figure 2Aiv) compared to the N-UNCD coated electrodes (Hejazi et al., 2020b). Furthermore, while N-UNCD coatings were found to delaminate and break following fiber bending, the B-CNW was firmly attached to the CF surface and survived a bending test without cracking, indicating better flexibility and mechanical stability. Therefore, B-CNW coated CFs are more suitable for building long-term closed-loop neural interfaces.

### Carbon Nanotube (CNT)-Based Fibers

Carbon nanotubes have attracted much attention since their emergence in the field of bioengineering due to their biocompatibility, and outstanding mechanical, electrical, chemical properties (Zestos et al., 2014; Vitale et al., 2015). It has been reported that both pristine and chemically functionalized CNT have a positive impact on neuronal growth (Smart et al., 2006). Due to their large effective surface areas and high conductivity, CNTs have been applied as coatings for improving electrode performance for neural stimulation and recording (Keefer et al., 2008; Motlagh et al., 2016; Kim et al., 2017).

Carbon nanotubes can be fabricated into microfibers or yarns via continuous spinning (Lee J. et al., 2019). CNT fibers fabricated via both wet and dry spinning have been applied as electrode materials for neural interfacing (Vitale et al., 2015). The diameters of the synthesized fibers are normally in a range between 5 and 50 μm, depending on the spinning parameters. CNT fibers typically exhibit excellent electrochemical properties for neural stimulation and recording. For example, Vitale et al. (2015) demonstrated the capability of CNT fibers fabricated by wet spinning for *in vivo* chronic neural stimulation and recording for 3 weeks (Figure 2Bi). In this work, they showed the successful use of CNT fiber electrodes with a diameter of 43 μm for deep brain stimulation in a Parkinson rat model (Figure 2Bii). The CNT fiber microelectrodes are suitable for chronic recording with no evidence of degradation of recording quality as observed from analysis of the temporal evolution of SNR (Figure 2Biii). After 6 weeks implantation, a four-fold reduction in the accumulation of astrocytes and a two-fold reduction in the expression of general microglia at the CNT fiber microelectrode site were measured. Expression of activated macrophages was found to be confined within approximately 50 μm from CNT fiber microelectrodes and to be more than two times less than at the PtIr site, where the region of activation extended to more than 150 μm (Figure 2Biv). CNT fibers were also demonstrated as more stable than PEDOT coatings after 97M vs. 43M cycles of pulsing tests and no significant biofouling was observed on the electrode surface after explantation (Vitale et al., 2015). In another work, Lu et al. (2019) used dry spun CNT fibers with a diameter between 5 and 20 μm. Their 20 μm fibers showed impedance of 279.96 ± 32.08 KΩ, which decreased to 41.95 ± 3.62 KΩ after nitric acid treatment. CIC also increased from 3.52 ± 0.15 to 5.04 ± 0.22 mC/cm2 after nitric acid treatment. Their fiber electrodes could record spontaneous activities from rat ventral posteromedial (VPm) nucleus of the thalamus and primary somatosensory cortex up to 5 months, and the tissue response was found much smaller than the PtIr controls. In this work, they showed that the CNT fibers are compatible with functional MRI, which allow the studies of the entire brain with simultaneous electrophysiology and MRI imaging.

Carbon nanotube fibers exhibit higher flexibility than CFs, which contributes to the minimal tissue response in chronic applications but introduces additional challenges during implantation. Several methods have been used to facilitate the implantation of CNTs. Vitale et al. (2015) used a polyimide shuttle and water soluble poly(ethylene oxide) coating to facilitate the electrode insertion. However, the stiffening shuttler increased the footprint during insertion, which was suggested to result in an enhanced neuronal loss around the CNT fiber microelectrodes observed from histology (Vitale et al., 2015). Lu et al. (2019) used a tungsten wire shuttle device to facilitate implantation, which has the same drawback of an increased insertion footprint. The authors then suggested the use of CFs as an alternative shuttle device as a means to reduce the insertion footprint. In a more recent study reported by Tang et al. (2020), the authors functionalized CNT fibers with a layer of calcium ion crosslinked sodium alginate. The functionalized fiber electrodes are rigid before implantation but become softer after insertion. A critical drawback of their design is that the diameter of the functionalized fibers increased from ∼36 to ∼190 μm after implantation. The significant swelling of the fibers could limit the application of this technology. The above shuttle methods and the use of stiffening agents can temporarily increase the electrode size and stiffness thus aggravating neural damage during implantation. To solve this issue, Vitale et al. (2018) inserted CNT fibers using a specially designed microfluidic device, which can apply a tension force onto the fibers that prevents the bending of electrodes without increasing the thickness or stiffness of the electrodes. Their method also allows the precise actuation of the electrode position with micro-scale accuracy.

### Graphene-Based Fibers

Graphene is another widely studied carbon-based material. In graphene, carbon atoms form hexagonal lattices in a 2-dimension plane and has a large effective surface area (Si and Song, 2018; Zeng et al., 2019). Many studies have indicated that graphene-based materials are biocompatible. For example, graphene produced by chemical vapor deposition with nanoscale dimensions has been shown to be friendly to several types of cells, viz., they enhance fibroblast adhesion and promote human mesenchymal stem cell (hMSCs) differentiation into bone cell (Kim et al., 2013; Wang et al., 2019). Mendonca et al. (2016) used healthy male Wistar rats for evaluating the nanotoxicity of reduced graphene oxide. In this study, the reduced graphene oxide produced minimal toxicological effects up to 7 days following tail vein injection. In another study, Rauti et al. (2016) investigated the effect of graphene oxide nanosheet on the synaptic signaling of cultured hippocampal neurons using patch clamp and fluorescence imaging. They showed the introduction of graphene oxide nanosheets down-regulated neuronal signaling but had no impact on cell viability.

Graphene-based materials can be applied for neural interfacing as a coating. Zhao et al. (2016) developed a graphene encapsulated copper microelectrode by CVD depositing a thin layer of graphene on 100 μm-diameter copper microwires. The coating largely eliminates the toxicity of copper, as indicated from both *in vitro* cell tests and *in vivo* histology studies. The extent of the gliosis from the graphene coated copper microwires was found comparable to that from the Pt microwires of the same diameter in terms of the upregulation level and zone size of activated microglia and astrocytes. However, for the graphene coated microwires, microglial and astrocytes tend to diffuse and distribute in a larger area away from the implant, which was suggested to be due to the antifouling surface of graphene. The coated electrodes have an impedance about 100 kΩ at 1 kHz and were used for both acute and chronic *in vivo* recording in rat hippocampus. Both LFPs and SU spikes could be recorded with the graphene coated electrodes for at least 4 weeks, and the performance was found comparable with conventional metal microwires. In this work, they also demonstrate the compatibility of these electrodes for use together with functional MRI.

The Melbourne group reported the fabrication of liquid crystal graphene oxide (LCGO) fibers for neural stimulation and recording (Figure 2Ci; Apollo et al., 2015). Such fibers are fabricated by first wet spinning liquid crystalline dispersion of graphene oxide into continuous fibers and then treating the fibers with hydroiodic acid. With a cylinder diameter of 50 μm, these LCGO fiber electrodes exhibit a CIC as large as 46 mC/cm2 and were shown to evoke neural activities in the explanted retinas. In this work, we also demonstrated SU recording in an acute study from feline visual cortex (Apollo et al., 2015; Figure 2Civ). Later, we compared the performance of electrodes fabricated using graphene fibers, CNT fibers and PtIr for chronic recording in an epilepsy rat model for 22 days. We showed that the graphene fibers outperformed all the other electrode materials, exhibiting the largest seizure SNR and only modest changes in impedance (Apollo et al., 2018). To further improve the electrode performance, Wang et al. (2019) suggested the use of a thin Pt coating as the current collector on the LCGO fibers, which decreases the fiber resistivity. The Pt coating was shown to improve both the CIC and CSC of the electrodes and decrease the electrochemical impedance. The maximum CIC reached 10.5 mC/cm2 for Pt coated graphene fiber electrodes with a diameter of 20 μm. The authors also demonstrated the stability of their electrodes after repetitive pulsing and cycling tests. Using a four-channel electrode array, they were able to record SU spikes with high SNRs in an acute study from rat motor cortex.

Zhao et al. (2020) used graphene fibers fabricated with a different technique for neural stimulation. In this work, their fibers were prepared through a one-step dimensionally confined hydrothermal process using suspensions of graphene oxide. The final diameter of their fibers is about 75 μm. The electrodes fabricated with the graphene fibers exhibit a CIC of 10.1 ± 2.25 mC/cm2 and were successfully used for deep brain stimulation in a behaving Parkinson rat model. As the graphene electrodes created little-to-no MRI artifact, they could study the activation pattern of stimulation using functional MRI imaging.

Park et al. (2017) developed a custom conductive polymer composite comprised of conductive polyethylene (CPE) and 5 wt% graphite for chronic *in vivo* recording and optical stimulation from cortex and hippocampus regions of mouse brain. This composite reduced electrode dimension and impedance, allowing for the integration of high density electrophysiology (6 electrodes), optical stimulation (a waveguide) and fluid delivery (two channels) within probes with diameters less than 200 μm, which are comparable to or smaller than those of silica fibers used for optogenetics. The flexibility and miniature footprint also enhanced the biocompatibility of the probes as indicated by stable long-term recordings of isolated SU action potentials as well as reduced glial response and BBB breach 3 months after implantation.

As with CNT fibers, graphene-based fibers exhibit superior flexibility (Figure 2Cii), which may create difficulties for electrode insertion. The Melbourne group developed a method to coat the 50 μm LCGO fibers with water-soluble sucrose microneedles to facilitate the electrode insertion (Figure 2Ciii). This method is also applicable to other flexible electrodes, such as CNT fibers. The damage from the sugar needle was found to heal over a 3-week duration and no sustained inflammatory response was observed (Apollo et al., 2018). However, both Wang et al. (2019) and Zhao et al. (2020) found that their graphene-based microfiber electrodes have sufficient mechanical robustness and sharpness to be inserted without any additional aid. Further research is required to study the chronic performance of graphene-based microfiber electrodes.

## Fabrication of Microfiber-Structured Electrode Arrays

**FIGURE 3:**
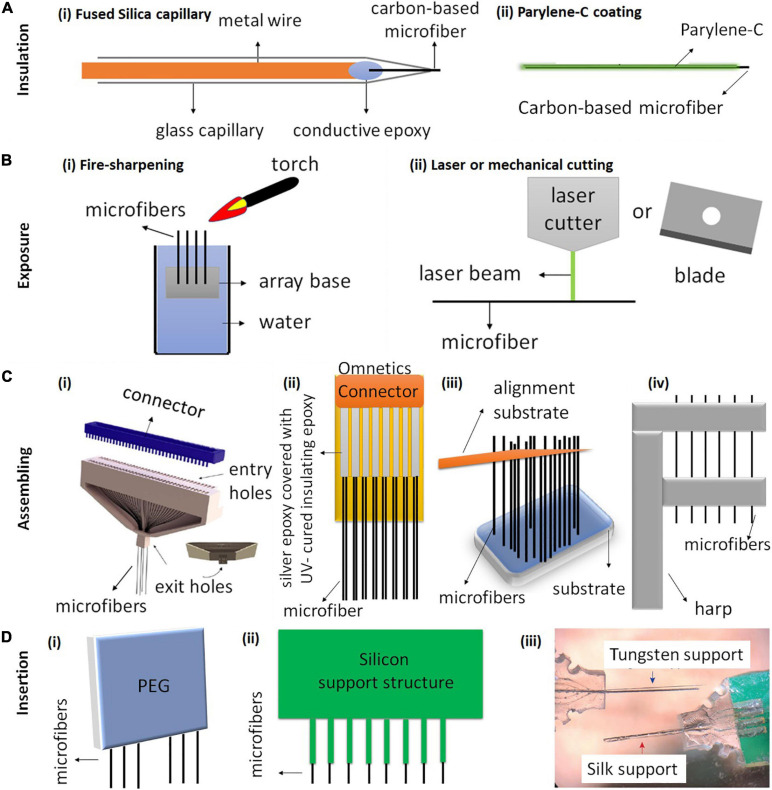
Schematic illustration of carbon-based microfiber electrode array fabrication. **(A)** The most common methods for insulating carbon-based fiber electrodes use either **(Ai)** fused silica capillary or **(Bii)** Parylene-C coating. **(B)** Methods for exposing the electrode tips include **(Bi)** fire-sharpening and **(Bii)** laser or mechanical cutting. **(C)** Four different techniques have been used to assemble single fibers into electrode arrays. **(Ci)** A 64-channel carbon fiber array fabricated using a 3D-printed block (gray) for aligning the microfibers. Reproduced from Guitchounts and Cox (2020) with permission from the copyright holder. **(Cii)** An electrode array with 16 CFs, 8 on each side. The CFs are attached on a PCB board using silver epoxy and the PCB board is soldered onto an Omnetics connector. Reproduced from Patel et al. (2020). **(Ciii)** of a threaded device during assembly. An alignment substrate separated from the device substrate is used to parallelize the 2.5 mm-long fibers. Reproduced from Massey et al. (2019) **(Civ)** Fibers are aligned using a harp-like structure fabricated by 3D printing and laser writing. Reproduced from Gillis et al. (2018). **(D)** Three different insertion methods for inserting CF arrays. **(Di)** A poly (ethylene glycol) (PEG) coating can facilitate the insertion of CF arrays by temporarily stiffening the fibers. PEG dissolves after application of sterile Ringer’s solution (Patel et al., 2015). **(Dii)** A silicon support structure with shanks and CFs secured within the shanks (Patel et al., 2015). **(Diii)** CF electrode arrays with tungsten supports and silk supports. Reproduced from Lee Y. et al. (2019).

A single microelectrode can only communicate with a single neuron or a small group of neurons. However, in both neuroscience research and clinical applications, simultaneous and precise communication with a larger population of neurons over a large area is often required (Obien et al., 2014). Therefore, MEAs with high channel counts and high densities are highly desirable. This section summarizes the techniques that have been developed to integrate individual carbon-based fiber electrodes into MEAs. The fabrication normally involves two procedures: (1) electrode insulation of the shank and exposure of the tip, and (2) electrode alignement and assembly. Figure 3 summarizes the methods used for insulation, tip exposure and fiber assembling. We also introduce the techniques for inserting arrays in this section.

### Electrode Insulation and Exposure

During fabrication, the microfibers are firstly insulated and then just the very tips are exposed for neural recording and stimulation. This is essential in order to reduce cross talk between electrodes and to maintain high spatial resolution.

There are several methods that have been used for electrode insulation. A common method is to insulate the fibers in a fused silica capillary (Figure 3Ai). However, the silica shaft normally has a diameter over 90 μm, which limits the chronic application of the electrodes due to the large implant footprints (Schwerdt et al., 2018). Therefore, thinner polymer coatings have been developed for insulation to reduce the overall diameter of the electrodes. Among different polymer materials, the most commonly used insulation is Parylene-C coating, which can be deposited on the electrode surface using a Parylene-C coater with thickness as thin as 1 μm (Figure 3Aii; Guitchounts et al., 2013; Guitchounts and Cox, 2020). Parylene-C is pinhole-free and chemically inert. It resists swelling in aqueous solutions and retains the flexibility of the microfibers (Tan and Craighead, 2010). Other polymer coatings that have been used for insulating carbon-based microfibers include Parylene-N, polystyrene-polybutadiene, poly(oxyphenylene), polyacrylonitrile, and polyethylacrylate (Jerome et al., 2001; Budai et al., 2007).

There are several different methods that have been used to expose the fiber tip. Fire sharpening is one commonly used method applied on fibers with Parylene-C coating (Figure 3Bi). In the process of fire sharpening, the fibers are firstly dipped into water with the other ends left exposed. Then, a flaming torch is passed over the water/air interface, removing the insulation whilst the sharpened fibers remain in the water (Guitchounts et al., 2013; Lee Y. et al., 2019; Guitchounts and Cox, 2020). The sharpening leads to the fiber tips having a cone shape, and it can produce a low electrode impedance to an acceptable range (around 1 MΩ for CFs) for extracellular recording (Guitchounts et al., 2013).

Different cutting methods have also been attempted and compared to expose carbon-based microfiber electrodes (Figure 3Bii). For example, both surgical scissors and razor blades were used to expose CF coated Parylene-C for neural recording. However, such mechanical cutting results in electrodes with varying impedances, often as high as 4 MΩ, which is unsuitable for neural recording (Guitchounts et al., 2013). Electrodes exposed using laser cutting exhibit a clean tip and excellent sidewall quality (Niino et al., 2016). Patel et al. (2020) reported that the arrays fabricated using laser cutting could lead to better chronic recording stability than those fabricated using blade cutting. The improved performance was suggested to be due to a better control and cleaner exposed tip surface with laser cutting. The Melbourne group has used laser cutting to expose the LCGO fiber electrodes coated with Parylene-C (Figure 2Di; Apollo et al., 2015). Our results showed that the laser cutting led to a brush-shape tip end, with enhanced effective surface area and surface oxidation, both of which contribute to the improved electrochemical properties of the electrodes (Apollo et al., 2015). Another cutting method to expose the ends uses cryo-sectioning (Massey et al., 2019). In this method, the electrodes are first embedded in a block of Tissue-Tek 4583 embedding compound and frozen to −80°C. The electrodes are then mounted into the cryotome held at −50°C and progressively shaved in 10 μm slices with a TiN-coated blade until the tips of fibers are exposed. The embedding compound is then thawed and thoroughly rinsed in deionized water.

### Electrode Alignment and Assembling

An ideal alignment and assembling method should be time-efficient and involve minimal manual handling. This method should allow the deposition of insulating materials onto individual fibers and enable electrode assembly with adjustable pitch and high electrode counts. Furthermore, the design of MEA can have an impact on the tissue response after implantation. Therefore, it is desired to assemble the microfibers on a smaller, lighter and softer substrate. These substrate materials are also required to be biocompatible and stable. Four fabrication examples are shown in Figure 3C. In all the examples, the positioning and alignment of the fibers are performed using molds, which have grooves or channels for constraining the movement of the individual fibers.

The first example of an assembling method for CF array fabrication was developed by Guitchounts et al. (2013) and Guitchounts and Cox (2020), in which they were able to fabricate CF arrays with up to 64 channels for chronic electrophysiology (Figure 3Ci). Briefly, the authors threaded the CFs through a 3D-printed plastic block, coated the fibers with Parylene-C, exposed them using fire sharpening and finally functionalized the fibers with PEDOT:TFB for chronic *in vivo* recording (Guitchounts et al., 2013; Guitchounts and Cox, 2020).

Figure 3Cii shows another design developed by Patel et al. (2015, 2020, 2016). They built a 16-channel CF array which is mounted on a PCB, with a connector soldered on the top of the array. The fibers from both sides of the array were attached using silver epoxy, which was then oven cured. In this device, fibers are spaced at a pitch of 132 μm. CFs were first cut to 1 mm long and then coated with approximately 800 nm thick of Parylene-C. After coating, the CFs were cut down to 500 μm in length and the tips were re-exposed using laser ablation. They demonstrated chronic neural recording and dopamine sensing by implanting the arrays in rat nucleus accumbens for 1 month. Additionally, electrodes were left in the tissue, sliced in place during histology and showed minimal tissue damage (Patel et al., 2020). Similarly, Schwerdt et al. (2017, 2018) aligned ten CF with individual lengths of 5.5 mm on a PCB using a glass substrate with trenches (250 μm pitch). The fibers were connected to the PCB using silver epoxy, which was later cured on a hot plate. The CF (50–200 μm long) were subsequently masked with photoresist. The fibers were finally insulated with Parylene-C deposition followed by lifting-off of photoresist masks with acetone to expose the CF tips.

A third example was developed by Massey et al. (2019), in which they fabricated 32 channel CF arrays using Si microfabrication and micro-assembling (Figure 3Ciii). In this device, fibers are spaced at a pitch of 38 μm, the smallest pitch reported so far for carbon-based fiber arrays (Massey et al., 2019). The authors suggest that the fabrication technique is scalable to a larger number of electrodes and allows for the potential future integration of microelectronics. They demonstrated acute recording using the arrays in rats.

Figure 3Civ shows the last example of a mold for fiber assembly, which was fabricated by 3D printing and laser writing (Gillis et al., 2018). Using this mold, CFs were placed in the harp-like structure to improve positioning and handling during subsequent steps. In this design, the alignment clips are 150 μm apart, therefore the density of the electrodes after assembling is relatively low. Another limitation is the number of channels, which is only four. To connect the arrays to other electronics, a polyimide lead was custom designed to serve as an interconnector between the electrodes and an Omnetics connector (Gillis et al., 2018). The connector was soldered to one end of the lead using a reflow oven, which is used primarily for the reflow soldering of surface mount electronic components to PCB. The other end of the lead was prepared for electrode bonding by rinsing it with isopropanol and spraying off the excess with nitrogen gas (Gillis et al., 2018). In this array, the electrodes were insulated using Parylene-C and exposed with fire sharpening. The authors also functionalized CFs with EIROF to improve the electrode properties. The arrays were then demonstrated for both acute stimulation and recording in the right-side tracheosyringeal nerve in zebra finches.

To summarize, the harp-structure assembling method (Figure 3Civ) resulted in a low-density electrode array (Gillis et al., 2018) while the device designed by Patel et al. (2015, 2020) (Figure 3Cii) has a higher channel count (16 channels). Both types of fabrication lead to arrays with pitch size above 132 μm. In comparison, the arrays developed by Guitchounts and Cox (2020) (Figure 3Ci) and (Massey et al., 2019; Figure 3Ciii) have higher electrode counts (32 or 64 channels), and the Massey array has lowest pitch size of 38 μm. The entire fabrication of the arrays by Guitchounts and Cox (2020) (Figure 3Ci) and the harp-structure assembly method (Gillis et al., 2018) takes about 2 h, but (Patel et al., 2020) and Massey et al. (2019) did not mention the fabrication time required. However, all designs involve manual steps. Techniques that can position and align the fibers automatically to facilitate the assembly process should be developed.

### Insertion Techniques

Insertion method is an important factor to be considered when fabricating high-density CF arrays. The use of additional supports for CF insertion was found to strongly depend on the fiber length (Patel et al., 2015; Massey et al., 2019). To determine the optimal length for reliable insertion, Massey et al. (2019) inserted CFs arrays into 0.6 w/w% agar gel which mimics many mechanical properties of the brain. The results showed that the longest fibers (3.5 mm in length) could not insert, while the fibers shorter than 3.5 mm could insert successfully. Those lengths could be variable once implanted *in vivo* as agar is not a perfect model for cortical tissue. Therefore, the authors suggested a practical upper bound of 2.5–3 mm for devices. CFs of longer lengths therefore require additional support which can provide them with sufficient mechanical stiffness and facilitate the insertion into deeper brain regions (Patel et al., 2015; Schwerdt et al., 2018; Lee Y. et al., 2019).

An ideal insertion technique should allow the insertion of CF arrays with high channel count without introducing acute or chronic tissue responses due to insertion damage. Patel et al. (2015) and Schwerdt et al. (2018) temporarily stiffened CFs tips using poly (ethylene glycol) (PEG) coating, which later dissolved with sterile ringer’s solution just above the brain surface (Figure 3Di). Patel et al. (2015) suggested that this method was suitable for insertion of arrays with only one or two rows of fibers, but difficult for inserting arrays with three or more rows (Patel et al., 2015). Patel et al. (2015) also demonstrated a second method, in which they used a silicon support structure consisting of small grooves for holding individual fibers (Figure 3Dii). The use of silicon support enabled insertion of arrays with three or more rows (Patel et al., 2015). In another work (Lee Y. et al., 2019), CF arrays were embedded within two different supporting materials, biodegradable silk fibroin coatings and non-degradable tungsten wires, to facilitate the insertion of CF into deeper brain regions (Figure 3Diii). The silk support structure dissolved approximately 2 days after implantation. Their result showed that electrodes with silk supports induced less reactive glial responses than that with tungsten supports.

## Remaining Challenges and Future Direction

Carbon-based microfiber electrodes provide advantages of minimal tissue response and improved resolution for neural stimulation and recording, compared to conventional electrodes fabricated using metal wires or silicon technologies. However, there are several remaining challenges that need to be addressed for their future wide use in both neuroscience research and medical applications.

The first urgent challenge is to construct MEAs using these fiber materials with high electrode count and density. The fabrication must be highly controllable, with high successful yield, minimal manual procedures and therefore minimal fabrication time. Although optical methods for recording neural activities have already made important contributions to studying neural activities, the existing imaging techniques are limited in terms of temporal resolution (Rector et al., 2009). The scattering of light in the brain and thermal sensitivity of brain tissue also limit the application of imaging techniques and many of them require the use of florescent proteins that create barriers in clinical translation (Hillman, 2007; Park et al., 2018). Compared with imaging, electrical recording can provide much higher temporal resolution (He et al., 2011). It is also possible to record deep from the brain using penetrating electrodes and the clinical translation is relatively easy (Im and Seo, 2017). However, to record from large populations of neurons and large brain areas, it is necessary to develop arrays with large electrode counts and densities. The highest electrode count from arrays fabricated using carbon-based fiber electrodes so far is only 64, which is smaller than many other electrode arrays. For example, the most widely used Utah arrays have 100 microelectrodes. One recently reported recording system, the Argo, is constructed with 65,536 recording channels (Obaid et al., 2020), which is suitable for *in vivo* research (Sahasrabuddhe et al., 2020). It is therefore important to develop novel technologies to scale-up the fabrication of carbon-based microfiber arrays. Most of the studies that have investigated inflammatory tissue responses from carbon-based microfibers were performed using single or very small numbers of electrodes. The influence of electrode count and density on the tissue response to array implantation also needs to be considered.

Second, there is a demand to improve the insertion technique to facilitate the implantation of these flexible fiber electrodes. Existing studies for inserting flexible carbon-based microfibers use either bio-dissolvable coatings (Apollo et al., 2015) or thick shuttle devices (Lee Y. et al., 2019). Both approaches increase the insertion footprints, which may lead to adverse tissue responses and therefore limit the chronic application of the fibers (Weltman et al., 2016). Many other insertion techniques that have been developed for other flexible implants may also be applicable to carbon-based microfibers. For example, a “sewing machine” has been developed and used to implant arrays with 64 shanks for minimal invasive neural recording (Hanson et al., 2019). The design of this insertion tool can minimize the overall insertion footprint, vasculature disruption and maximize the number and anatomically distribution of targeted electrodes (Hanson et al., 2019).

Last, future neural interfaces will require the devices to perform multiple functions to obtain the most detailed and comprehensive information from the nervous system, while having the capacity to simultaneously modulate neural activities with the greatest precision and control. Compared to electrical modalities, to stimulate and record from neurons optically or chemically can provide additional information and flexibility (Hong and Lieber, 2019). For instance, the integration of optical fiber electrodes, which bi-directionally transmit light between separate sites (even at a distance of several micrometers), will enable simultaneous electrophysiology and optical imaging or neural stimulation via optogenetics (Miyamoto and Murayama, 2016). The use of optogenetic manipulation will make it possible to control neural activity with cell-type and projection selectivity, which will advance our understanding of specific circuit activity and behaviors (Miyamoto and Murayama, 2016). Chemically, neurotransmitter measurements and stimulation can be another useful add-on function in neural interfaces (Sung et al., 2020). Neurotransmitters play an important role in neural communications (Niyonambaza et al., 2019). They are involved in psychological processes such as learning and memory, and their pathologies are correlated with many psychiatric or neurological disorders such as Parkinson’s disease, schizophrenia, and Alzheimer’s disease (Si and Song, 2018). Many of these carbon-based microfibers have been demonstrated to sense neurotransmitters such as dopamine, serotonin and glutamine (Fang et al., 2017). However, many of these developments are still in the primary stages.

Despite all of these challenges, carbon-based microfiber electrodes hold the promise for next generation neural interfaces. With minimal tissue response and the capacity for high-resolution neural stimulation and recording, carbon-based microfibers are expected to serve as the core technologies in the closed-loop devices that can communicate reliably and efficiently with neurons for an adequately long period. The developed technologies will benefit both basic neuroscience and medical research by deepening our understanding of neural functions and advancing novel therapy development, which will ultimately improve our quality of living.

## Author Contributions

All authors listed have made a substantial, direct and intellectual contribution to the work, and approved it for publication.

## Conflict of Interest

SP is a shareholder in iBIONICS, a company developing a diamond based retinal implant. SP and DG are shareholders and public officers of Carbon Cybernetics Pty Ltd., a company developing diamond and carbon-based medical device components. The remaining authors declare that the research was conducted in the absence of any commercial or financial relationships that could be construed as a potential conflict of interest.
